# Research of a Novel Ultra-High Pressure Sensor with High-Temperature Resistance †

**DOI:** 10.3390/mi9010005

**Published:** 2017-12-25

**Authors:** Guo-Dong Zhang, Yu-Long Zhao, Yun Zhao, Xin-Chen Wang, Xue-Yong Wei

**Affiliations:** State Key Laboratory for Manufacturing Systems Engineering, Xi’an Jiaotong University, Xi’an 710049, China; zhangguodong@stu.xjtu.edu.cn (G.-D.Z.); zhaoyun@stu.xjtu.edu.cn (Y.Z.); wangxinchen@stu.xjtu.edu.cn (X.-C.W.); seanwei@mail.xjtu.edu.cn (X.-Y.W.)

**Keywords:** ultra-high pressure sensor, silicon-on-insulator (SOI) piezoresistive element, small size, high-temperature resistance, microelectromechanical systems (MEMS) technology

## Abstract

Ultra-high pressure measurement has significant applications in various fields such as high pressure synthesis of new materials and ultra-high pressure vessel monitoring. This paper proposes a novel ultra-high pressure sensor combining a truncated-cone structure and a silicon-on-insulator (SOI) piezoresistive element for measuring the pressure up to 1.6 GPa. The truncated-cone structure attenuates the measured pressure to a level that can be detected by the SOI piezoresistive element. Four piezoresistors of the SOI piezoresistive element are placed along specific crystal orientation and configured as a Wheatstone bridge to obtain voltage signals. The sensor has an advantage of high-temperature resistance, in that the structure of the piezoresistive element can avoid the leakage current at high temperature and the truncated-cone structure separates the piezoresistive element from the heat environment. Furthermore, the upper surface diameter of the truncated-cone structure is designed to be 2 mm for the application of small scale. The results of static calibration show that the sensor exhibits a good performance in hysteresis and repeatability. The temperature experiment indicates that the sensor can work steadily at high temperature. This study would provide a better insight to the research of ultra-high pressure sensors with larger range and smaller size.

## 1. Introduction

Ultra-high pressure sensors are of great importance in many special civil and military applications, such as high pressure synthesis of new materials, pressure calibration of diamond anvil cell, shock wave propagation, explosive detonation, and high-speed impact. To date, numerous ultra-high pressure sensors based on various sensing principles—including piezoresistive, piezoelectric, capacitive, and optical fiber—have been developed [1]. The piezoelectric pressure sensor must be combined with a charge amplifier because of its high output impedance and it can only measure the dynamic pressure [2]. The capacitive sensor possesses large nonlinearity, for the restriction of sense principle [3]. Optical-fiber-grating sensor’s structure is complicated, which makes output signal conditioning complex and sensor packaging difficult [4]. The piezoresistive sensor has been mainly used to measure the ultra-high pressure because of low output impedance, large output signal, excellent accuracy and good dynamic property. In literature, there are many papers proposing numerous ultra-high pressure piezoresistive sensors with different structures. The design concepts of those structures can be divided into two main broad categories such as (a) the measured pressure is directly applied to the sensitive element; and (b) the sensitive element is separated from the measured pressure by elastic elements. As for the first category, piezoresistive materials including carbon, ytterbium, and manganin are made into sensitive films that can subject the ultra-high pressure uniformly without any drastic stress concentration. Nevertheless, the piezoresistive curve of the carbon pressure gage is nonlinear [5]. Ytterbium is a kind of material in which phase transformation will take place at high pressure. This limits the measuring range of the ytterbium sensor [6]. The manganin gage offers a low piezoresistive coefficient and its accuracy is affected by the transverse tensile strain effect [7]. In the second category, many elastic element structures with different materials are designed. Among those sensors, sensitive elements and elastic elements of some sensors are integrated together which allows them to perform with high sensitivity and precision, such as the silicon piezoresistive sensor [8,9,10,11,12]. However, since the silicon structure is weak in strength, their measuring range is often no more than 300 MPa. Therefore, using high strength material as an elastic element can effectively increase the measuring range of the sensor. Fu [13] considered a piezoresistive pressure transduce which used high intensity alloy film as initial sensing element to endure the measured pressure and silicon oil to translate the pressure to piezoresistive sensing element. Its measuring range is 0–400 MPa. Zhao [14] explored an ultra-high pressure sensor based on autofrettaged cylinder structure made of the spring steel of 60Si2CrVA and silicon flat chip with an inverted cup structure. Its measuring range is 0–1 GPa. Zhao [15] studied an ultra-high pressure sensor combining a cylindrical elastic body made of the stainless steel of 17-4PH and a micro silicon-on-insulator (SOI) solid piezoresistive chip. Its measuring range is 0–2 GPa. All these sensors mentioned above lay a foundation for the research of ultra-high pressure sensors with larger range and better accuracy. 

In general, ultra-high pressure environments are often accompanied by high temperature, such as explosive detonation [16], high pressure synthesis of new materials [17], and ultra-high pressure vessels [18]. High temperatures will affect the measurement accuracy of the sensor. However, many researchers focus more on the measuring range than on high-temperature resistance of the sensor. This research proposes an ultra-high pressure sensor combining a truncated-cone structure and an SOI piezoresistive element. It exhibits a large measuring range up to 1.6 GPa, high-temperature resistance, small size, and good static performance. The sensor reported here could be beneficial to the research of increasing the measuring range and further improving the performance of high-temperature resistance of ultra-high pressure sensors.

## 2. Materials and Methods

### 2.1. Structure of the Sensor

**Figure 1:**
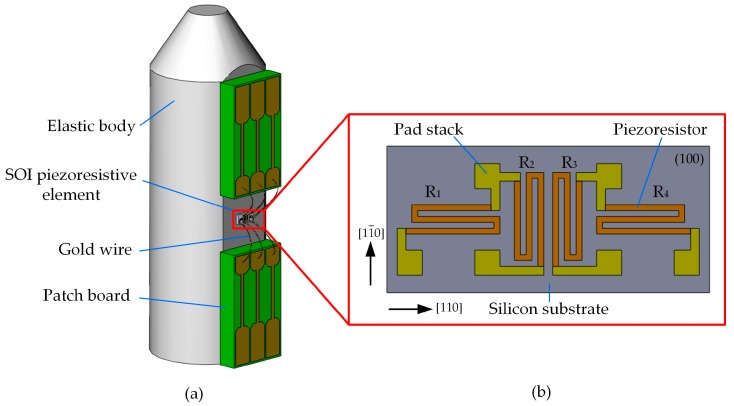
Schematic of the sensor: (**a**) structure of the sensor; (**b**) layout of the silicon-on-insulator (SOI) piezoresistive element.

As shown in Figure 1a, the ultra-high pressure sensor is composed of an elastic body, an SOI piezoresistive element, and two patch boards. The elastic body has two sections: one is called truncated-cone section and the other is cylinder section. The truncated-cone section was designed to reduce the ultra-high pressure to a value that can be detected by the SOI piezoresistive element. The SOI piezoresistive element and patch boards were attached to the same side of the cylinder section.

The piezoresistive element was fabricated on an SOI wafer whose buried SiO_2_ layer can avoid the leakage current at high temperature [19]. Four equivalent piezoresistors with sheet resistances of 10 Ω/□ were located in the (100) crystal plane which has a small crystal alignment deviation. In order to obtain the maximum and equivalent piezoresistance coefficent, we let piezoresistors be parallel to specific orientation by microelectromechanical systems (MEMS) technology, as Figure 1b shows. The beam lead method of the pad stacks was used to solve the problem of connection between the inside and the outside of the piezoresistive element at high temperature. Titanium was chosen to form ohmic contact with *p*-type silicon because of its low specific contact resistivity (SCR). The theoretical contact resistance of a single piezoresistor is about 8.89 mΩ, which is beneficial to the work stability of the piezoresistive element. Aurum was chosen as the third layer of the beam lead to contact with the external leads. Platinum was used as second layer of the beam lead to prevent the formation of Ti-Au intermetallic compound. The patch boards were connected with the pad stacks through gold wires. A full Wheatstone bridge widely used in MEMS sensors was formed in an external circuit for signal read out yielded by the piezoresistors [20,21].

### 2.2. Working Principle of the Sensor

The ultra-high pressure sensor was developed based on the equivalent transfer of a force in the elastic limit range and the piezoresistive effect of silicon. The work principle is described as follows.

**Figure 2:**
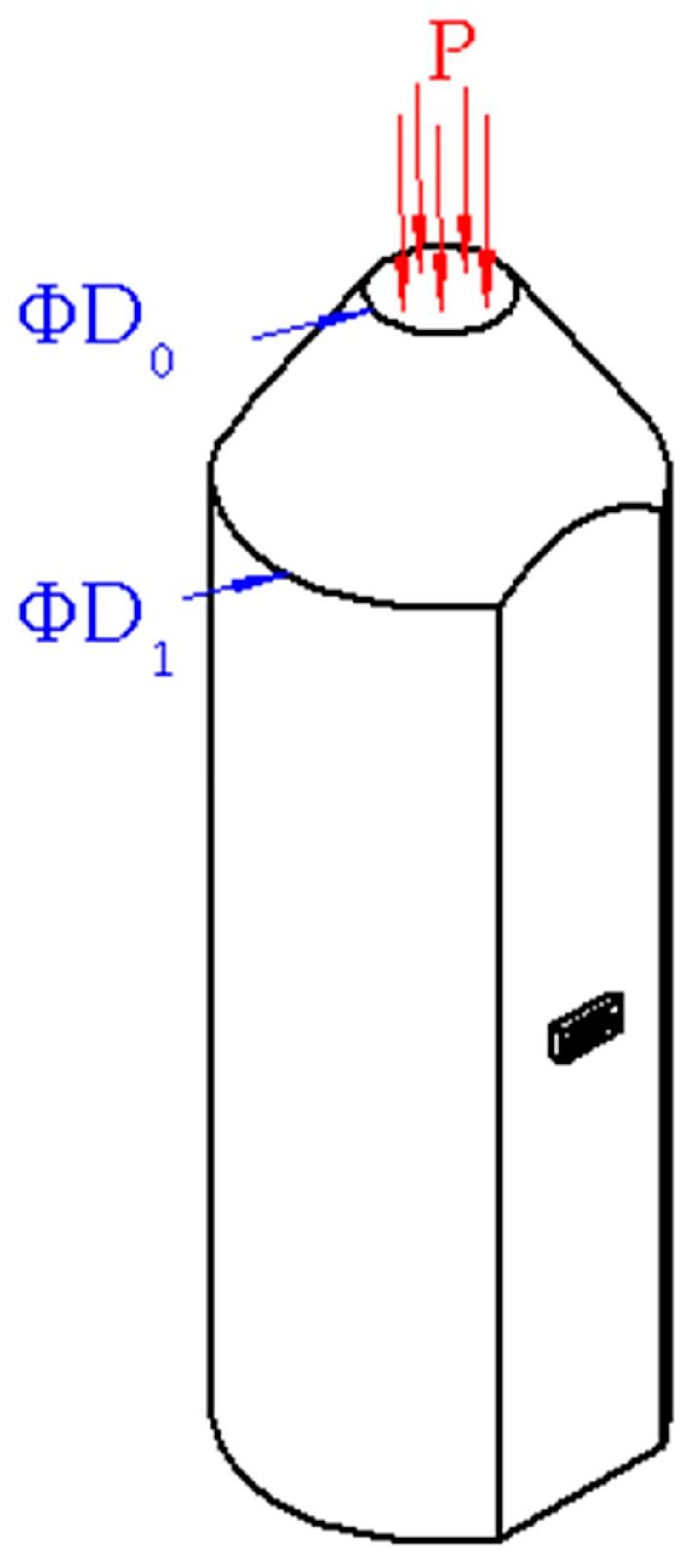
Work principle of the sensor.

As shown in Figure 2, when an ultra-high pressure is applied on the upper surface of the truncated-cone section, the cylinder section will produce a strain. According to the equivalent transfer of a force within the elastic limit range, we have the equation

(1) $$\varepsilon_{1}=\frac{P\cdot A_{0}}{A_{1}\cdot E_{1}}$$

where *ε*_1_ is the strain of the place where the sensitive element was attached, *P* is an ultra-high pressure applied on the upper surface of the truncated-cone section, *A*_0_ and *A*_1_ are area of upper surface of the truncated-cone section and cross sectional area of the place where the sensitive element was attached, respectively, *E*_1_ denotes elastic modulus of the elastic body.

As for piezoresistors of the piezoresistive element, the relative change ratio of resistivity along the resistor stripe can be written as [22]

(2) $${\left(\frac{\Delta \rho }{\rho_{0}}\right)}_{l}=\tau_{l}\sigma_{l}+\tau_{t}\sigma_{t}$$

where *τ_l_* and *σ_l_* are longitudinal piezoresistive coefficient and longitudinal stress along the direction parallel to the resistor stripe, respectively, *τ_t_* and *σ_t_* denote transverse piezoresistive coefficient and transverse stress along the direction perpendicular to the resistor stripe, respectively.

In this research, equivalent piezoresistors were arranged in (100) crystal plane and along [110] and [1$\bar{1}$0] crystallographic orientations. According to Equation (2), the relative change ratio of *R*_1_ and *R*_4_ can be expressed as

(3) $$\frac{\Delta R_{1}}{R_{1}}=\frac{\Delta R_{4}}{R_{4}}=-\frac{1}{2}\pi_{44}\sigma_{2}$$

where the minus sign indicates that resistances of *R*_1_ and *R*_4_ decrease when there is only a principal stress along the [1$\bar{1}$0] crystallographic orientation, *π*_44_ is shear piezoresistive coefficient of *p*-type silicon, *σ*_2_ denotes the principal stress along the [1$\bar{1}$0] crystallographic orientation. Similarly, we can obtain the relative change ratio of *R*_2_ and *R*_3_

(4) $$\frac{\Delta R_{2}}{R_{2}}=\frac{\Delta R_{3}}{R_{3}}=+\frac{1}{2}\pi_{44}\sigma_{2}$$

where the plus sign indicates that resistances of *R*_2_ and *R*_3_ increase when there is only a principal stress along the [1$\bar{1}$0] crystallographic orientation.

Based on the principle of Wheatstone bridge, output voltage of the bridge can be written as

(5) $$V=\frac{\Delta R}{R}\cdot V_{0}$$

where *V*_0_ is the voltage of constant voltage source. On substituting Equation (4) into Equation (5) and solving for *σ*_2_,

(6) $$\sigma_{2}=\frac{2V}{V_{0}\cdot \pi_{44}}$$

Then we have

(7) $$\varepsilon_{2}=\frac{2V}{V_{0}\cdot \pi_{44}\cdot E_{2}}$$

where *ε*_2_ is the principal strain along the [1$\bar{1}$0] crystallographic orientation. *E*_2_ denotes the elastic modulus of silicon.

In this study, the piezoresistive element was attached to the elastic body with M-Bond 610 and [1$\bar{1}$0] crystallographic orientation of the piezoresistive element is parallel to axial direction of the elastic body. Suppose that

(8) $$\varepsilon_{1}=\varepsilon_{2}$$

Under above situation. On substituting Equations (1) and (7) into Equation (8) and solving for *P*,

(9) $$P=\frac{2}{\pi_{44}}\cdot \frac{A_{1}\cdot E_{1}\cdot V}{A_{0}\cdot E_{2}\cdot V_{0}}$$

It can be noted from the Equation (9) that the measured pressure is proportional to the output voltage of Wheatstone bridge. That is to say, as long as we know the output voltage of Wheatstone bridge, we can work out the measured pressure. However, in practical applications, we calculate the measured pressure through the calibration curve of the sensor.

### 2.3. Design Details of the Sensor

As we know, silicon has a strain limit of 0.003 [19]. Using this restrictive condition and the hypothesis shown in Equation (8), measuring range of the sensor is limited by following inequation

(10) $$P\leq {\left(\frac{r_{1}}{r_{0}}\right)}^{2}\cdot E_{1}\cdot \frac{\varepsilon_{m}}{k}$$

where *r*_0_ and *r*_1_ are radii of upper and lower surface of the truncated-cone section, respectively, *ε_m_* is the strain limit of silicon, *k* is safety factor of the strain limit. It can be observed that the measuring range of the sensor depends on the radius ratio of lower and upper surface of the truncated-cone section and the elastic modulus of the elastic body. Moreover, the sensor must work in the elastic range. Therefore, the following inequation needs to be met

(11) $$P\leq \sigma_{s}$$

where *σ_s_* is yield strength of the elastic body. Only when Equations (10) and (11) are satisfied at the same time can the sensor work properly. In other words, the measuring range of the sensor depends on the radius ratio of lower and upper surface of the truncated-cone section and elastic modulus and yield strength of the elastic body.

Tungsten alloy was used to produce the elastic body. As listed in Table 1 [23], it offers high Young’s modulus and yield strength, which can help to increase the measuring range of the sensor. In addition, tungsten alloy also has a high melting point and low coefficient of thermal expansion (CTE), which can help the sensor work at high temperature.

Many factors should be considered to design dimensions of the sensor. According to our processing capacity and practical applications, the minimum value of *r*_0_ can be set to 1 mm. In order to take full advantage of high yield strength of the elastic body, we consider that

(12) $$\sigma_{s}\leq {\left(\frac{r_{1}}{r_{0}}\right)}^{2}\cdot E_{1}\cdot \frac{\varepsilon_{m}}{k}$$

Given that *k* = 5, we obtain

(13) $$r_{1}\geq 2.78\;\mathrm{mm}$$

**Figure 3:**
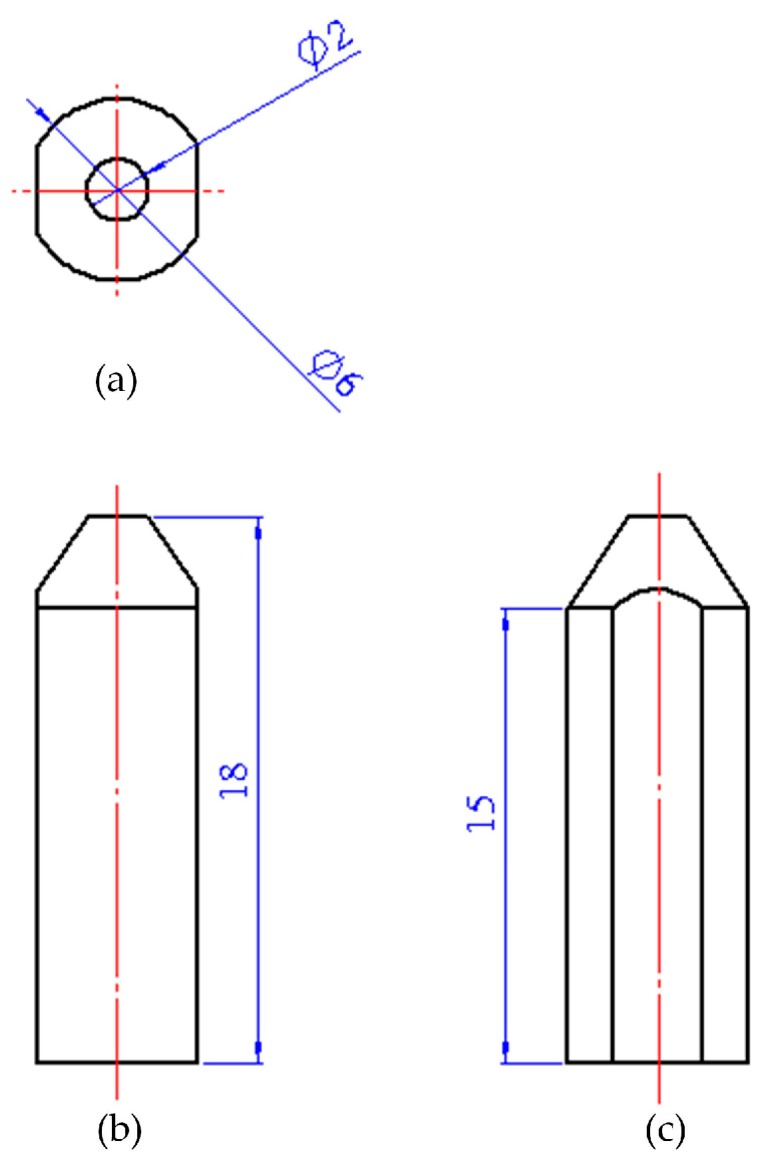
Three views of the elastic body: (**a**) top view; (**b**) front view; (**c**) left view.

In order to obtain a small size sensor, we let *r*_1_ be equal to 3 mm. The rest sizes of the sensor are shown in Figure 3. Under the above conditions, the sensor proposed in this study has a measuring range of up to 1.6 GPa.

### 2.4. Analysis of High-Temperature Resistance

High-temperature resistance of the sensor can be attributed to the following two aspects. First, the piezoresistive element was fabricated on an SOI wafer by MEMS technology. Second, high temperature of the measured environment was dissipated mostly by the elastic body.

The SOI technology introduces a buried oxide layer between the top silicon and the silicon substrate. The piezoresistors of the piezoresistive element is separated from the silicon substrate by the oxide layer to avoid the leakage current between the piezoresistors and the silicon substrate at high temperature, thereby improving high-temperature resistance of the piezoresistive element. In addition, the high temperature experiment showed that the resistance values of the piezoresistors can keep relatively stable for two hours at 250 °C [19].

**Figure 4:**
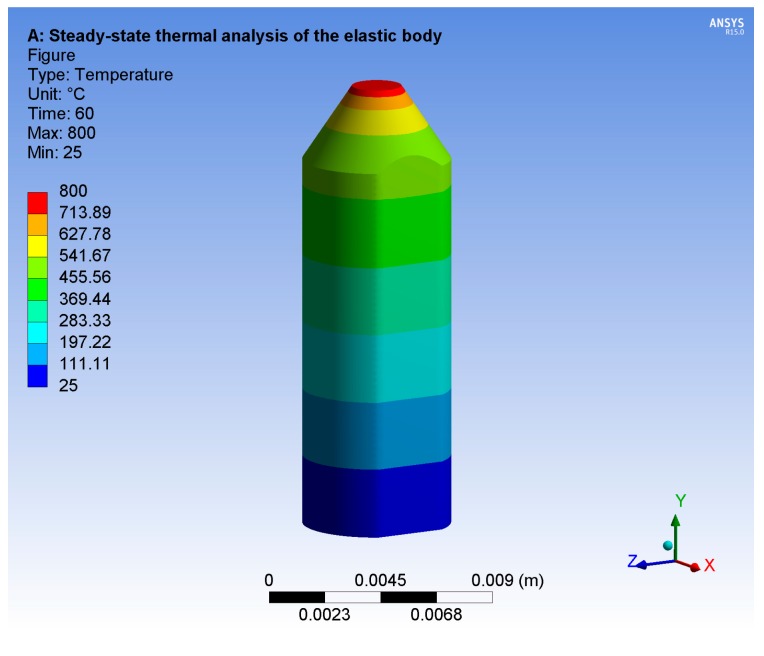
Steady-state thermal analysis of elastic body.

The heat conduction of the elastic body was analyzed by ANSYS 15 (Ansys, Inc., Canonsburg, PA, USA). A thermal load of 800 °C was applied on the upper surface of the elastic body, and the steady-state temperature distribution of the elastic body is presented in Figure 4. Especially, the temperature of the place where the piezoresistive element was attached is approximately 250 °C. This result indicates that the working temperature of the sensor is as high as about 800 °C (the upper surface of the elastic body is exposed to the measured environment).

The above design considerations can not only increase the working temperature of the sensor, but also reduce the impact of temperature on the accuracy of the sensor.

### 2.5. Fabrication of the Sensor

**Figure 5:**
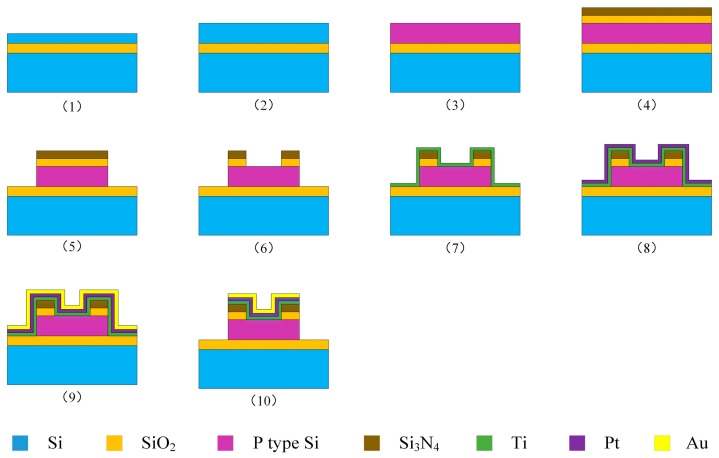
Fabrication process of the SOI piezoresistive element.

**Figure 6:**
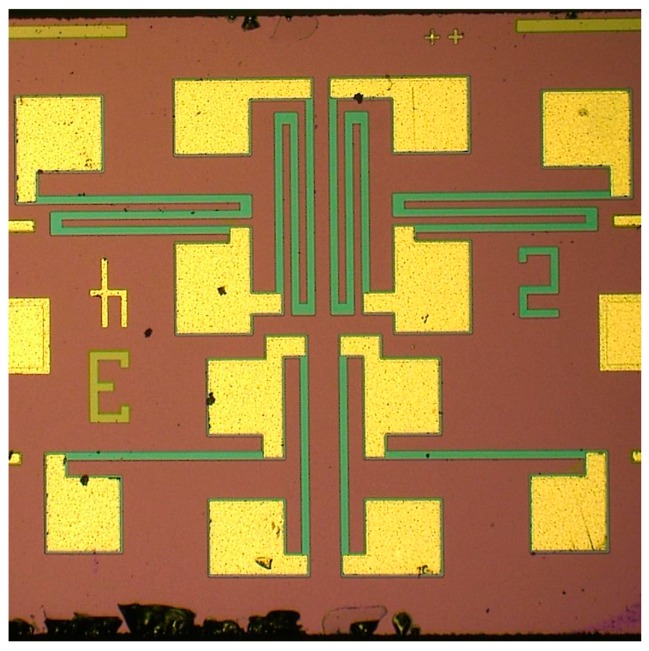
SEM photograph of the SOI piezoresistive element.

The SOI piezoresistive element was fabricated by MEMS technology. Its fabrication process is illustrated in Figure 5: (1) clean the SOI wafer with dilute HF solution to remove the native oxide; (2) increase the thickness of top silicon layer by low pressure chemical vapor deposition (LPCVD) to ensure the sensitivity of piezoresistive element; (3) dope boron ions into top silicon layer and make annealing treatment for forming *p*-type silicon which offers high piezoresistive effect and good temperature resistance; (4) form SiO_2_ layer by thermal oxidation for improving the stability of measurement circuit layer and produce Si_3_N_4_ layer as a stress matching layer by LPCVD; (5) etch piezoresistors by reactive ion etch (RIE); (6) etch contact holes for exposing *p*-type silicon; (7) sputter Ti, Pt, and Au successively; (8) corrode the extra metal layer for forming electrodes; (9) keep 30 min under 550 °C in vacuum for good ohmic contact between Ti and *p*-type silicon. Finally, a single piece of piezoresistive element was obtained after dicing. Figure 6 shows a SEM photograph of the fabricated SOI piezoresistive element with a size of 1.8 × 1.6 × 0.2 mm^3^. There are two groups of measuring circuits with resistances of 1 KΩ and 2 KΩ, respectively, on every SOI piezoresistive element. In this study, the resistors of 2 KΩ were used to form a Wheatstone bridge.

The elastic body was fabricated with 93 tungsten alloy by mechanical processing. Its outline dimensions are presented in Figure 3. The roughness of the cutting face on both sides of the elastic body was required to be 3.2 μm for better attaching the piezoresistive element.

**Figure 7:**
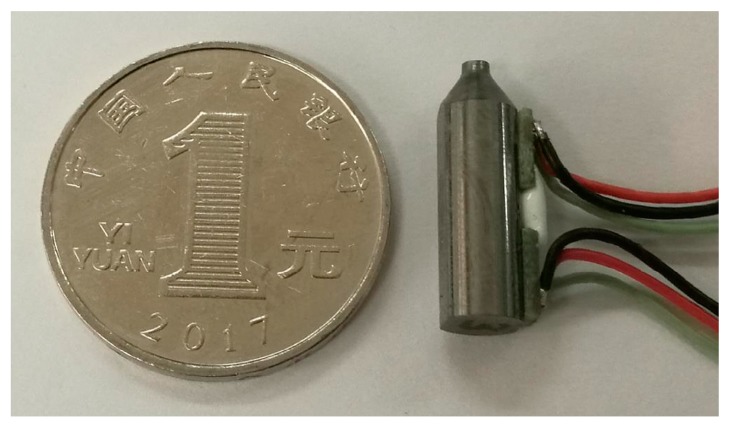
Photograph of the fabricated sensor.

The SOI piezoresistive element was attached on the cutting face with M-Bond 610 and its [1$\bar{1}$0] crystallographic orientation is parallel to axial direction of the elastic body. Then, two patch boards were symmetrically arranged at both sides of the piezoresistive element. The measuring circuit with 2 KΩ on the piezoresistive element was connected with the proximal side of the patch boards through gold wires. A Wheatstone bridge was formed by leading wires from the other side of the patch boards. The SOI piezoresistive element, gold wires, and welding points were coated with high temperature-resistance pastern. Figure 7 illustrates a photograph of the fabricated sensor.

### 2.6. Static Calibration

**Figure 8:**
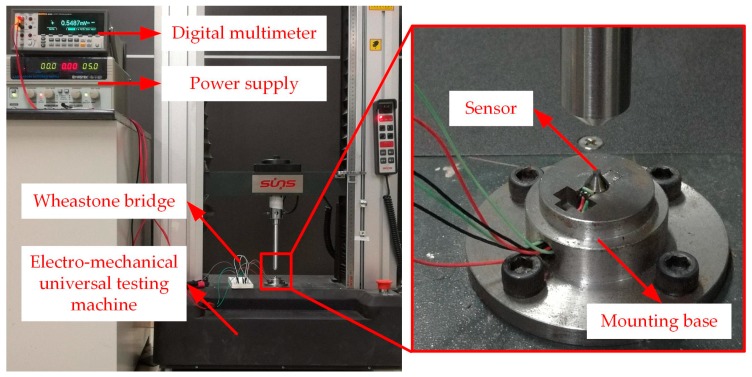
Experimental setup for static calibration.

Static calibration test was performed to determine the static characteristics of the sensor. In this study, measuring range of the sensor is 0–1.6 GPa. Accordingly, the force exerted on the upper surface of the elastic body should be 0–5 KN. Static calibration test was operated on an electro-mechanical universal testing machine which could apply standard force for a certain time. During the test, the upper surface of the elastic body was applied from 0 N to 5 KN with an interval of 500 N and each interval would be maintained for 30 s. The measuring circuit was excited by 5V DC and the output signals were recorded by high accuracy and resolution precision digital multimeter. A diagram of the experimental setup is shown in Figure 8.

### 2.7. Temperature Experiment

The temperature experiment is important to show the high-temperature resistance of the sensor. Due to the limitations of experimental conditions, we have preliminarily tested the temperature characteristics of the sensor under no load condition. According to the steady state thermal analysis shown in Figure 4, the working temperature of the piezoresistive element is about 250 °C when the sensor is at the upper limit of its working temperature (it is needed to emphasize that only the upper surface of the elastic body is exposed to the measured environment when the sensor is in the working state). Then, we tested the temperature characteristics of the sensor at 200 °C on the basis of our experimental conditions. 

**Figure 9:**
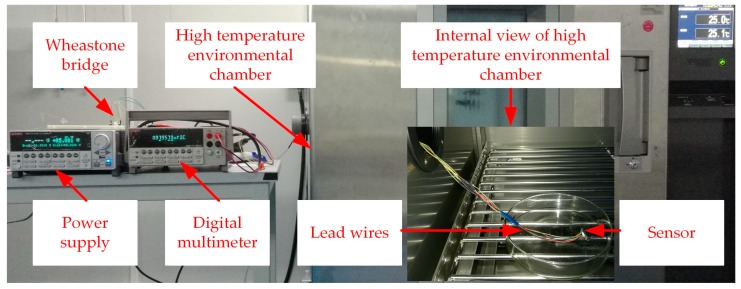
Experimental setup for temperature experiment.

As shown in Figure 9, the sensor was placed in high temperature environmental chamber which can provide standard temperature from 0 to 200 °C for a certain time. The measuring circuit was excited by 5V DC and the output signals were recorded by a digital multimeter.

## 3. Results and Discussions

### 3.1. Static Calibration

**Figure 10:**
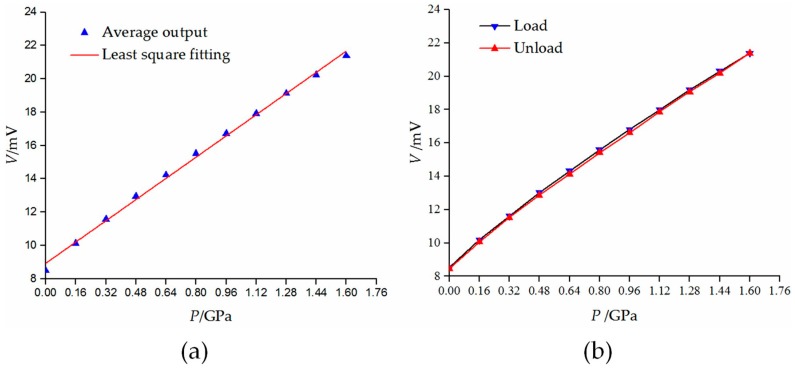
Static characteristics of the sensor: (**a**) theoretical work line; (**b**) hysteresis characteristics curve.

The static calibration test for the developed sensor was performed five times and the average output data were recorded in the Table 2. As Figure 10a shows, the theoretical work line of the sensor is obtained by the least square method with these data. It takes the form

(14) V(mV)=8.933+7.955P(GPa)

where *P* is an ultra-high pressure applied on the upper surface of the truncated-cone section, and *V* is output voltage of the digital multimeter.

In order to evaluate static characteristics of the sensor, four indicators are given based on the theoretical work line.

Hysteresis error can be calculated as follows

(15) $$H=\frac{\Delta y_{\max}}{\left(y_{\max}-y_{\min}\right)}\times 100\%$$

where *H* represents hysteresis error, Δ*y*_max_ is the sensor’s biggest output deviation between loading and offloading process under a series of input values, *y*_max_ and *y*_min_ are maximum and minimum output values of the sensor. Substituting the experimental data of Table 3 into Equation (15), we obtain *H* = 1.45%. The hysteresis characteristic curves of the sensor are shown in Figure 10b.

Repeatability can be obtained through Equations (16) and (17)

(16) $$S_{i}=\sqrt{\frac{\sum_{j=1}^{n}{\left(y_{ij}-\bar{y_{i}}\right)}^{2}}{n-1}}$$

(17) $$R=\frac{3S_{\max}}{y_{\max}-y_{\min}}\times 100\%$$

As for a certain test cycle, assuming the number of measuring points is *m*, and for each measuring point, the output value has been measured for n times. Thus, S_i_ denotes standard deviation of each measuring point, *y_ij_* is the *j*th (*j* = 1 − *n*) measured value of the *i*th (*i* = 1 − *m*) measuring point, *$\bar{y}$_i_* is the average value of the measured values of *i*th measuring point. *R* stands for repeatability error, *S*_max_ represents the biggest standard deviation among *S_i_*. On calculating *R*, *R* = 0.28%.

Linearity can be represented as

(18) $$L=\frac{\Delta_{\max}}{y_{\max}-y_{\min}}\times 100\%$$

where Δ_max_ is the biggest deviation value between the sensor’s theoretical work line and its practical one, *L* denotes the linearity error. After the calculation, we have *L* = 3.44%.

The accuracy of the sensor is a comprehensive indicator that reflects the sensor’s static performance. Combining the above-calculated values, we obtain

(19) $$A=\sqrt{H^{2}+R^{2}+L^{2}}=3.74\%$$

### 3.2. Temperature Experiment

Thermal zero drift test was conducted at 25 °C and 200 °C for one hour, respectively. The zero output was read every 15 minutes. The output data were recorded in the Table 4.

Thermal zero drift can be represented as

(20) $$\gamma =\frac{\left|{\bar{y}}_{0}\left(T_{2}\right)-{\bar{y}}_{0}\left(T_{1}\right)\right|}{Y_{FS}\left(T_{1}\right)\left(T_{2}-T_{1}\right)}\times 100\%$$

where $\bar{y}$_0_(*T_i_*) (*i* = 1, 2) is zero average output in one hour at temperature *T_i_*. *Y_FS_*(*T*_1_) denotes full scale output of the sensor at temperature *T*_1_. Let *T*_1_ = 25 °C and *T*_2_ = 200 °C in Equation (20). It can be observed from Table 2 that the full scale output of the sensor at temperature 25 °C is 21.388 mV. Then we have *γ* = 0.015%°C^−1^.

Zero temperature coefficient reflects the change of zero outputs with the temperature. It can be expressed as [17]

(21) $$\alpha =\frac{\Delta y_{om}}{Y_{FS}\cdot \Delta T}\times 100\%$$

where Δ*y_om_* is maximum variation of zero outputs in a certain range of temperature, *Y_FS_* is full scale output of the sensor, Δ*T* denotes the range of temperature. Zero outputs of the sensor at different temperatures are given in Table 5. On calculating *α*, *α* = 0.016% °C^−1^.

Zero time drift at a certain temperature can preliminarily characterize the working stability of the sensor at that temperature. From Table 4, the zero time drift at 200 °C is calculable as [17]

(22) $$D_{t}=\frac{y_{\max}-y_{\min}}{\Delta t}=0.039\;\mathrm{mV}/h$$

where *D_t_* is zero time drift, *y_max_* and *y_min_* are maximum and minimum zero outputs in a certain time range at 200 °C, respectively, Δ*t* denotes the range of time.

## 4. Conclusions

This paper proposes a novel structure combining a truncated-cone section and an SOI piezoresistive element for ultra-high pressure sensor with the measuring range of 0–1.6 GPa. The structure design, work principle, and characteristic analysis were presented in detail. The analysis result shows that the sensor can achieve the ultra-high pressure measurement at high temperature up to 800 °C in theory. By the calibration, we obtained the performance indexes of the sensor such as linearity of 3.44% of full scale (FS), repeatability of 0.28% of FS, hysteresis of 1.45% of FS, and accuracy of 3.74% of FS. These performance indexes can basically meet the general demands of ultra-high pressure measurement. In addition, we conducted the zero temperature experiment which indicated preliminarily that the sensor can work steadily at high temperature. This research has a positive reference value to other researches of the ultra-high pressure sensor with higher range and smaller size. In future work, we will continue to study high-temperature resistance of the sensor proposed in this paper. Furthermore, we will design a new micromachined structure utilizing the principle of stress concentration to be integrated with the piezoresistive element for measuring the ultra-high pressure with high accuracy.

## Figures and Tables

**Table 1:** Material parameters of tungsten alloy.

Young’s Modulus (GPa)	Yield Strength (GPa)	Melting Point (°C)	Coefficient of Thermal Expansion (1 × 10^–6^/°C)
345	1.6	3410	4.5

**Table 2:** Average output data.

Force (N)	Corresponding Pressure (GPa)	Average Output Data (mV)
0	0	8.495
500	0.16	10.125
1000	0.32	11.571
1500	0.48	12.949
2000	0.64	14.234
2500	0.8	15.511
3000	0.96	16.712
3500	1.12	17.917
4000	1.28	19.126
4500	1.44	20.243
5000	1.6	21.388

**Table 3:** Average output data.

Pressure (GPa)	Loading (mV)	Unloading (mV)
0	8.532	8.458
0.16	10.182	10.068
0.32	11.622	11.520
0.48	13.028	12.870
0.64	14.324	14.144
0.80	15.598	15.424
0.96	16.804	16.620
1.12	17.980	17.854
1.28	19.188	19.064
1.44	20.299	20.186
1.60	21.380	21.388

**Table 4:** Zero output at 25 °C and 200 °C.

Time (min)	Zero Output at 25 °C (mV)	Zero Output at 200 °C (mV)
0	8.505	7.947
15	8.518	7.934
30	8.526	7.912
45	8.513	7.938
60	8.507	7.951

**Table 5:** Zero outputs at different temperatures.

Temperature (°C)	Zero Output (mV)
25	8.513
50	8.532
75	8.545
100	8.372
125	8.121
150	7.968
175	7.942
200	7.936
